# Glances at the Hospitals

**Published:** 1901-01-19

**Authors:** 


### GLANCES AT THE HOSPITALS.

### THE DORSET COUNTY HOSPITAL.

THE total receipts, excluding legacies, were £2,325, and the total expenditure was £2,439. The legacies amounted to £150. The average weekly number of in-patients was 32 the average cost per week per head was a little over 23s. At the close of 1899 the balance due to the treasurer was £147, and this was chiefly due to an increase of £91 in the expenditurre, and to the non-payment of dividend on railway stock. The hospital contained 28 patients on January 1st, 1899, and 289 were admitted during the year, making a total of 317. Of these 150 were discharged cured, 71 relieved, 29 were made out-patients, 21 for other reasons, 18 died, and 28 remained under treatment. The total number of out-patients was 1,062, and of these 25[illegible] were cured, 546 were released, 25 were made in-patients, 81 discharged for other reasons, 3 died, and 151 were under treatment at the end of the year. The medical tables are well arranged, and convey all essential information. There were 105 operations performed during the year. Of these 71 were discharged cured, 15 relieved, and 2 died subsequent to the operation. Anæsthetics were administered on 122 occasions, chloroform being the agent 38 times, ether 37 times, a mixture of chloroform and ether 9 times, nitrous oxide and ether 12 times, and nitrous oxide alone 26 times.

### THE HOSPITAL FOR SICK CHILDREN, NEWCASTLE-ON-TYNE.

THE income amounted to £3,307 and the expenditure to about the same sum, but a sum of £135 was owing to the bank on January 1st, 1899 and is included in the expenditure. The ordinary subscriptions reached £788 and the workmen's contributions came to £196. A "shelter" for the open-air treatment of consumption has been erected, and the whole of the cost thereof has been defrayed by Mrs. John Collins, of Michel-Dever. There were 772 in-patients and 4,526 out-patients. The average cost of each in-patient was £3 11s. 9d. The statistics are carefully compiled, and in all cases show the results of treatment. In the medical wards 334 patients were treated, with a mortality of 11·3 per cent. In the surgical wards there were 438 patients under treatment, with a mortality of 4·5 per cent. The total number of operations performed was 851 and the mortality was 2·2 per cent., but, of course, the total number contained a great many minor operations. Of the 772 in-patients, 492 were discharged cured, 113 relieved, 48 not improved, 58 died, and 58 remained under treatment. The average death-rate was 7·5 per cent.

### THE ANCOATS HOSPITAL.

THIS hospital owes to the bank a sum of £643. The chairman, in his speech at the annual meeting, said that the support received by the hospital "was not in proportion to the amount of work it did. The institution was carried on very economically, but at present the expenditure was at the rate of £400 or £500 in excess of the income." This is a deplorable state of affairs, and either the public must subscribe more freely, or the usefulness of the hospital will be crippled. Time will show which of these will happen. The workpeople's subscriptions amounted to £360. The medical and surgical tables show the number of patients suffering from the various diseases, and the number of deaths is also given; but the table of operations only gives the operations and the numbers, and says nothing of results. Altogether there were 141 major operations, and in several cases the results would have been instructive. During the year 808 accidents were attended to; 2,281 patients were seen at their own homes; there were 3,850 out-patients and 1,279 in-patients. We hope the tables will show an improvement in the report for 1900, and that the operation statistics will have a note appended showing the nature of the anæsthetic used.

### THE ROYAL SOUTHERN HOSPITAL, LIVERPOOL.

IT would seem that the income of this hospital amounts to £9,574, and that there is now an adverse balance of £4,140, but much of the latter was incurred by structural alterations and additions. We failed to find any statement of the average cost per bed occupied, or of the average cost per head, or of the average stay of the patients in the hospital. The medical and surgical cases are carefully tabulated. Of 41 cases of pneumonia, 24 recovered, 2 were relieved, 12 died, and 3 were still under treatment. There were 21 cases of typhoid fever and only 1 death. The table of operations performed is equally clear. There were 21 cases of ovariotomy, and of these 13 recovered, 3 were relieved, 1 died, and 4 were under treatment. Herniotomy was performed 28 times, with

25 recoveries, 1 relieved, 2 deaths. The total number of medical cases was 1,062; of surgical cases 1,384; and of operations 891. It is not stated what anæsthetic is used. There were 225 deaths in the hospital, but 46 occurred within 24 hours of admission. The out-cases numbered 10,233. This hospital contains a ward specially appropriated for tropical diseases, and 170 cases were treated in that ward during 1899. Details of these cases will be found in the Report issued by the Liverpool School of Tropical Diseases.

